# P-1436. Patient dissatisfaction increases the odds of inconsistent influenza vaccination completion following the onset of the COVID-19 pandemic

**DOI:** 10.1093/ofid/ofae631.1610

**Published:** 2025-01-29

**Authors:** Olivia Man, Jack W McHugh, Jeremy Young, Laurie Wilshusen, Lacey Hart, Tripp Welch, John C O’Horo, Douglas Challener

**Affiliations:** Mayo Clinic, Rochester, Minnesota; Mayo Clinic, Rochester, Minnesota; Carleton University, Ottawa, Ontario, Canada; Mayo Clinic, Rochester, Minnesota; Mayo Clinic, Rochester, Minnesota; Mayo Clinic, Rochester, Minnesota; Mayo Clinic, Rochester, Minnesota; Mayo Clinic, Rochester, Minnesota

## Abstract

**Background:**

The COVID-19 pandemic has underscored challenges with patient adherence to medical directives, particularly vaccine acceptance. While sociodemographic factors have been linked to non-COVID-19 vaccine hesitancy, the relationships between patient experience, the COVID-19 pandemic, and adherence to routine vaccinations are uncertain.

**Figure 1:**
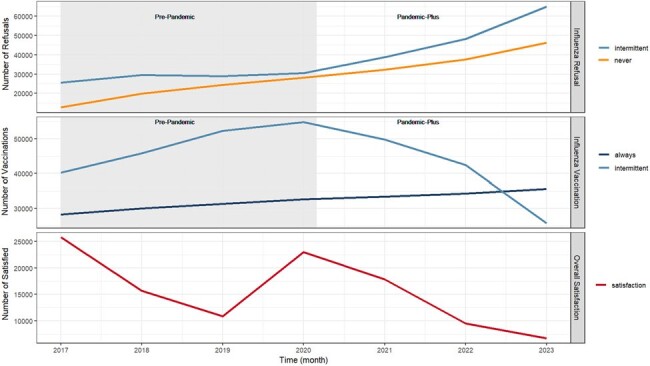
Yearly influenza vaccine refusal, influenza vaccine administrations, and overall patient satisfaction since establishing care with a primary care provider from 2017-2023. Panel A (Influenza Refusals): the number of vaccine refusals among individuals who are Intermittently Vaccinated and Never Vaccinated for seasonal influenza since establishing care with a primary care provider over time. Panel B (Influenza Vaccinations): the number of vaccines administered over time since establishing care with a primary care provider among those who are Always Vaccinated and Intermittently Vaccinated for seasonal influenza. Panel C (Overall Satisfaction): the number of patients who indicated that they were maximally satisfied with their overall care following any interaction with the healthcare system over time.

**Methods:**

We retrospectively analyzed influenza vaccination records of adult primary care patients in Olmsted County, Minnesota. Vaccination history was examined during two phases: Pre-Pandemic (1/1/2017—2/28/2020) and Pandemic-Plus (3/1/2020—12/31/2023). Vaccination status was defined as “always vaccinated” (AV), “never vaccinated” (NV), or “intermittently missed years of vaccinations” (IV) for seasonal influenza. Patient dissatisfaction was determined if patients negatively ranked their overall quality of care on standard patient experience surveys. We explored the relationship between influenza vaccination rates, healthcare engagement, and patient satisfaction.

**Figure 2:**
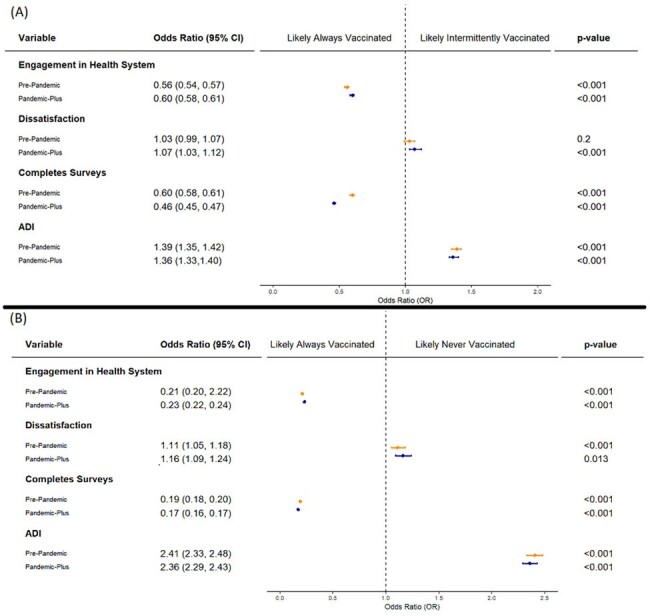
Multinomial logistic regression of patient satisfaction on vaccination status across the phases of the COVID-19 Pandemic, adjusted for engagement in the health care system, participation, and area of deprivation index (ADI) for patients that were (A) intermittently vaccinated compared to always vaccinated and (B) never vaccinated comparted to always vaccinated.

The “Pre-pandemic phase” refers to influenza immunization data and patient satisfaction data prior to March 2020. The “Pandemic-plus phase” refers to immunization data and patient satisfaction data from March 2020 to January 2024.

Low ADI ranking refers to areas with a low level of deprivation, whereas high ADI ranking refers to areas with a high level of deprivation. Individuals were considered low if they came from an area with a state ADI ranking from 1 to 5 and were considered high if they came from an area with a state ADI ranking from 6 to 10.

**Results:**

From 1/1/2017 to 12/31/2023, 21% of individuals were AV (n=36,632), 52% were IV (n=90,776), and 27% were NV (n=46,418) for seasonal influenza. Post-pandemic onset, 7% (n=3,556) of the Pre-Pandemic AV and 32% (n=16,710) of the Pre-Pandemic IV never received another influenza vaccine. In the Pandemic-Plus phase, the IV and NV had a higher likelihood of disengagement with the healthcare system [odds ratio (OR) (95% CI): 1.59 (1.55, 1.64); 4.21 (4.09, 4.33)], residing in areas of greater deprivation [1.58 (1.53, 1.62); 1.99 (1.94, 2.05)], and self-identifying their race as Black [2.29 (2.09, 2.51); 3.29 (3.01, 3.60)] compared to the AV. After adjusting for healthcare engagement and area of deprivation, dissatisfaction OR increased from 1.03 (0.99,1.07) Pre-Pandemic to 1.07 (1.03,1.12) in the Pandemic-Plus phase among IV compared to AV. Similarly, the OR of dissatisfaction rose from 1.11 (1.05, 1.18) Pre-Pandemic to 1.16 (1.09, 1.24) in the Pandemic-Plus phase among those who were NV compared to those AV.

**Conclusion:**

Higher healthcare dissatisfaction was observed among those inconsistently adherent to vaccination recommendations. Targeted efforts to enhance patient satisfaction may boost vaccination rates.

**Disclosures:**

**All Authors**: No reported disclosures

